# Biocontrol of Occurrence Ochratoxin A in Wine: A Review

**DOI:** 10.3390/toxins16060277

**Published:** 2024-06-17

**Authors:** Slaven Zjalic, Ksenija Markov, Jelena Loncar, Zeljko Jakopovic, Marzia Beccaccioli, Massimo Reverberi

**Affiliations:** 1 Department of Ecology, Agronomy and Aquaculture, University of Zadar, Trg kneza Viseslava 9, 23000 Zadar, Croatia; jloncar@unizd.hr; 2 Faculty of Food Technology and Biotechnology, University of Zagreb, Pierottijeva 6, 10000 Zagreb, Croatia; ksenija.markov@pbf.hr (K.M.); zjakopovic@pbf.hr (Z.J.); 3 Department of Environmental Biology, Sapienza University of Rome, Piazzale Aldo Moro 5, 00185 Rome, Italy; marzia.beccaccioli@uniroma1.it

**Keywords:** ochratoxin A, wine, biocontrol

## Abstract

Viticulture has been an important economic sector for centuries. In recent decades, global wine production has fluctuated between 250 and almost 300 million hectoliters, and in 2022, the value of wine exports reached EUR 37.6 billion. Climate change and the associated higher temperatures could favor the occurrence of ochratoxin A (OTA) in wine. OTA is a mycotoxin produced by some species of the genera *Aspergillus* and *Penicillium* and has nephrotoxic, immunotoxic, teratogenic, hepatotoxic, and carcinogenic effects on animals and humans. The presence of this toxin in wine is related to the type of wine—red wines are more frequently contaminated with OTA—and the geographical location of the vineyard. In Europe, the lower the latitude, the greater the risk of OTA contamination in wine. However, climate change could increase the risk of OTA contamination in wine in other regions. Due to their toxic effects, the development of effective and environmentally friendly methods to prevent, decontaminate, and degrade OTA is essential. This review summarises the available research on biological aspects of OTA prevention, removal, and degradation.

## 1. Introduction

Archaeological finds indicate that viticulture began in the Neolithic period, around 7000 BC. Some authors believe that the first wine was produced in China, while others believe that this fermented beverage is related to rice wine rather than *Vitis vinifera* wine. In the period of 7000–4000 BC, intentional fermentation of *Vitis vinifera* grapes is documented in archaeological sites in Georgia, Azerbaijan, Armenia, Iran, and Turkey [1]. The transition from the Caucasian region to Western Europe took a very long time. In ancient Greek and Roman culture, wine was a prized beverage used for various purposes, including medicine. The importance of wine is attested to by the existence of gods associated with wine in various cultures: Osiris in Egypt, Dionysius in Greece, and Bacchus in Rome [2]. Ancient wine was different from the wine we know today. The technology of winemaking has evolved over the centuries to today’s production [3]. According to the International Organisation of Vine and Wine (OIV), an intergovernmental organization, global wine production in recent decades has exceeded 250 million hectoliters (mhl) with peaks near 300 mhl. The main producers are European countries, with Italy, France, and Spain accounting for around 50% of global wine production. However, wine production is also important in other regions of the world. The top ten wine producers include the USA, Australia, Argentina, Chile, and South Africa, while Chinese wine production has declined in recent years and is no longer in the top 10. In 2022, the global wine export value amounted to EUR 37.6 billion, and the largest wine-producing countries are also among the largest exporters [4]. Fluctuations in annual wine production are, as with all crops, dependent on changing climatic conditions. Climatic changes could have a major impact on wine production in the near future, and some traditional wine-growing regions could become less suitable for wine production. An example of this is South Africa, which, according to the OIV report, recorded a slight decline in wine-growing areas for eight consecutive years in 2022 as a result of the droughts between 2015 and 2017 [4]. Climate changes with a rise in temperature and possible periods of drought followed by heavy rainfall could have an impact on wine production but could also increase the risk of contamination of wine with the mycotoxin ochratoxin A. Generally, multiple preliminary models depict a scenario where numerous areas will experience increased contamination with mycotoxin-producing fungi across various crops. Examples include the increased risk of mycotoxin contamination in wheat (trichothecenes) and maize (aflatoxins) as a result of climate change [5,6]. According to a recent report, increased carbon dioxide concentrations in the Mediterranean region may increase the risk of OTA contamination in wine [7]. Furthermore, under warmer climate scenarios, *Aspergillus niger* populations may prevail over *A. carbonarius* as they are well adapted to higher temperatures and drier conditions. OTA production may also be increased under a climate change scenario for *A. westerdijkiae*, which is thermophilic, at least compared to other “black” Aspergillia [8].

Ochratoxin A (OTA) is a mycotoxin produced by some species of the genera *Penicillium* and *Aspergillus. P. verrucosum* is considered among the most important species for contamination of food and feed in temperate climates, while *A. ochraceus*, *A. carbonarius,* and *A. niger* are more important in areas with higher temperatures [9]. In addition, *A. carbonarius* is the main cause of contamination of wine [10]. OTA was first isolated from *A. ochraceus* in 1965 and named after the producing species [11]. OTA has various toxic effects on humans and animals, with the kidneys being the main target. It has been reported to have nephrotoxic, immunotoxic, teratogenic, hepatotoxic, and carcinogenic effects on animals [12,13]. A carcinogenic effect has been demonstrated in animals but not in humans, which is why the International Agency for Research on Cancer has classified OTA in Group 2B, a possible human carcinogen [14]. Some authors [15,16] report that OTA is a probable cause of nephropathies and urothelial tumors in humans, while other authors have linked OTA to the endemic Balkan nephropathy, BEN [12,17]. In their review of epidemiologic studies on OTA exposure and adverse health effects in humans, ref. [9] concluded that while nephrotoxicity in animals is well documented, the results in humans are inconclusive. BEN is now associated with aristolochic acid and not with OTA [18]. Chemically, OTA is a stable molecule that can persist in food and feed due to its resistance to common food processing treatments such as heating or fermentation [19,20]. In addition, OTA is persistent in humans and has a rather long half-life in blood; also, it can be present in animal tissue and human breast milk [21,22,23,24]. Due to its toxicity, the European Commission has limited the concentration of OTA in food; the limit for wine is 2 ng mg^−1^ [25]. This regulation is no longer in force; it was replaced by EC Regulation 2023/915 on 25 April 2023, but the limit remained the same [26].

Contamination with OTA has been reported in corn, barley, wheat, nuts, dried fruits, smoked and dried fish, coffee grains, grapes, wine, beer, dried meat products, and cheese [9,27]. Cereal grains are the main source of daily intake of OTA by humans [28]. The contribution of wine to the daily intake of OTA depends on the dietary habits of the consumer. According to [29], wine could contribute significantly to daily OTA intake in some populations, up to 15%, ref. [30] reported that at the 31st meeting of the Codex Alimentarius Commission’s Committee on Food Additives and Contaminants, wine was cited as the second most important source of daily OTA intake after cereals. In more recent publications, various values are given for the same country in different years [10]. Although the exact contribution of wine consumption to the daily intake of OTA cannot be exactly calculated, it certainly contributes in some population groups. The World Health Organization recommends reducing the presence of mycotoxins in food and feed as much as possible [31], and this also applies to wine.

Climate change will affect all areas of agriculture, including wine production [32]. The rise in atmospheric temperature, erratic rainfall patterns, and the increase in extreme climatic events such as drought, storms, and heavy rainfall could affect not only productivity but also food safety. Environmental conditions could favor the spread of pathogens, including mycotoxigenic ones, to areas where they were not previously present and/or increase their occurrence in areas where they are already present [33]. With regard specifically to the risk of contamination of wine with OTA, climate change could strongly favor the occurrence of OTA. Temperature is one of the most important factors influencing the contamination of wine with OTA, as discussed previously and in the following chapter. The increase in temperature could lead to both a greater frequency of wine contamination and a higher concentration of OTA in wine in areas already considered at risk of OTA contamination, as well as creating a more favorable environment for wine contamination in areas previously considered at low(er) risk. Heavy rainfall, hail, and storms, especially in the late veraison phase, could damage the berries and favor fungal invasion and OTA production. These scenarios indicate that even more attention must be paid to plant protection in the future. The control of plant pathogens relies on the use of chemicals, but their use is constantly being restricted by legislation worldwide due to their harmful effects on the environment and human and animal health. Many of the chemicals used in plant protection have been banned in recent decades, particularly in EU countries [34]. Biocontrol agents are seen as a viable and more environmentally friendly alternative to conventional pesticides [35,36,37,38]. This paper provides an overview of the methods used to decontaminate and control OTA occurrence in wine through low environmental impact methods.

## 2. OTA in Wine

The presence of OTA in wine was first reported in 1996 [38]. In the following 10–15 years, various papers were published reporting the presence of OTA in wine in different countries around the world. Table 1 lists some of the publications investigating the presence of OTA in wine in different countries. As Europe is the largest producer of wine, it should come as no surprise that most of the research has been conducted in European countries. Even though these publications found the presence of OTA in wines from all over the world, in most cases, the concentration was (far) below the EU limits (Table 1). The highest OTA concentration in wine, 9.2 µg L^−1^, was reported by [39] for a wine from southern Italy. The overall results show that the presence of OTA is influenced by the type of wine and its geographical origin and, obviously, by seasonality. In general, red wine is more sensitive to OTA contamination, followed by rose wine and white wine [5,12,38,40,41]. For example, ref. [12] showed in their analysis of 420 wine samples of worldwide origin that 25% of the white wines examined had a detectable OTA concentration, 40% of the rose wines, and 54% of the red wines. Furthermore, the highest concentration was found in red wine (3.31 ng L^−1^), followed by rosé (2.38 ng L^−1^) and white wine (1.36 ng L^−1^). This is in line with the results of other authors [30,42,43,44,45].

**Figure 1:**
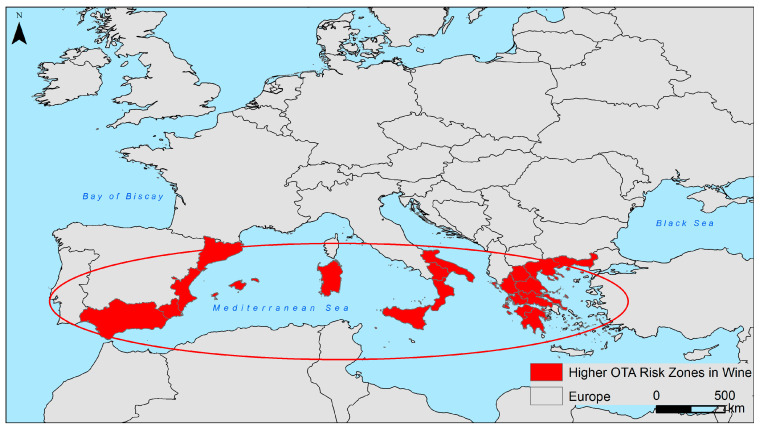
European regions considered at higher (circled in red) and high (painted in red) risk of wine contamination with ochratoxin A [12,30,42,43,44,46,47,48].

The occurrence of OTA in wine is related to the geographical location of the vineyards. Battilani et al. [30] reported that in Europe, the amount of OTA depends on the latitude of production; the lower the latitude, the higher and more frequent the occurrence of OTA in wine. Other authors also reported a higher occurrence of OTA in wines from southern regions [12,42,43,44,46,47,48]. According to these authors, the regions with the highest risk of OTA contamination in wine are southern Italy, south-eastern Spain, and parts of Greece and Turkey (Figure 1). For the rest of the world, such a correlation between latitude and OTA occurrence is not known, but it is generally assumed that wines from regions with higher temperatures, such as the Mediterranean, are more frequently contaminated with OTA than wines from regions with more temperate climatic conditions [10].

Other grape products, such as grape juices and dried grapes, have also been found to be contaminated with OTA [5,12,30,47,49,50].

**Table 1:** Presence of OTA in different types of wine in different parts of the world.

Country	Type of Wine	OTA Detected/Total Samples	Samples over the EU Limit (2 ng mL^−1^)	Source
Argentina	Red	4/47	3	[51]
	Red	136/136	0	[52]
Brazil	Red	18/26	0	[53]
	White	7/17	0	
Chile	Red	28/869	0	[54]
	White	6/319	0	
China	Red	183/183	0	[55]
	White	40/40	0	
Croatia	Red	7/7	0	[42]
	White	4/7	0	
			
	Red	6/6	0	[56]
	White	8/10	0	
France	red grape most	11/37	0	[57]
Greece	Red	58/78	0	[49]
	White	40/62	0	
	Sweet	6/10	0	
			
	Red	33/104	1	[58]
	White	55/118	0	
	Sweet	7/10	0	
Italy	Red	14/96	yes *	[46]
			
	Red	553/735	22	
	White	128/290	0	[43]
	Rose	69/75	4	
	Desert	18/28	0	
			
	Red	29/20	0	[48]
	Sweet	55/55	0	[59]
Poland	Red	49/53	yes *	[60]
Portugal	Red	9/35	1	[61]
	White	3/25	0	
South Africa	Red	15/15	0	[62]
	White	9/9	0	
Spain	Red	34/140	2	[50]
	White	6/60	1	
	Sweet	186/188	18	[40]
USA	Red	28/31	2	[63]
	White	7/10	0	

* The authors do not state the number of samples in which the OTA concentration is above the EU limits, but only the range of the minimum and maximum concentrations found.

## 3. Factors That Influence OTA Presence in Wines

OTA is synthesized on grape berries and contaminates the wine during winemaking. The crucial period for OTA contamination is between early veraison and harvest [30,64]. Conidia of ochratoxigenic fungi may be present on the berries, but their ability to penetrate the berries is low unless the berry is damaged, and wounds caused by biotic or abiotic factors favor their penetration [65]. One of the causes of berry bursting is excessive watering, either by irrigation or by rainfall, before harvest [66]. Insects, especially *Lobesia botrana*, the grape berry moth, can contribute significantly to OTA contamination by damaging the barriers [65,67,68]. As already reported in the previous chapter, climatic conditions, in particular air temperature and humidity, are the most important factors for OTA contamination of wines. Temperature and humidity influence the growth of all molds. The optimal temperature and a_w_ for the growth of *A. carbonarius* is 25–30 °C and an a_w_ value of 0.930 to 0.987, depending on the strain. However, the optimal temperature for OTA production within the same strains was different, 15–20 °C [69]. Other authors reported that the optimal temperature and water activity for *A. carbonarius* growth and optimal OTA biosynthesis may be different [70]. The grape variety and its intrinsic genetic characteristics (i.e., a compact grape shape is more exposed to OTA contamination than a loose one), as well as the state of ripeness, can also influence the occurrence of OTA in wine [44,45,71]. A positive correlation between the concentration of soluble solids, pectin content, total anthocyanins, and OTA content is reported. In laboratory tests using berries from three different varieties at different stages of ripeness, *A. carbonarius* inoculated onto the Muscat Italia variety showed greater OTA production in the early ripening stage when pH and sugar content were lower [45]. Passamani et al. [70] also found that lower pH contributes to OTA biosynthesis.

The role of oxidative stress in the regulation of OTA synthesis has been demonstrated [72]. In another experiment, prooxidants, but also some antioxidants, enhanced the OTA production of *A. carbonarius* [73]. This is confirmed by [45]. They observed that some antioxidants showed a positive correlation with OTA production, while for some others, the correlation was negative, suggesting that a structure-dependent signal is involved in the inhibition.

Another factor that can influence OTA production is carbon dioxide. Experiments with grape-based growing media have shown that higher CO_2_ concentrations stimulate OTA production. The higher CO_2_ concentration (1000 ppm) shortened both the lag phase of fungal growth and increased OTA production. In some fungal strains, the combined effect of higher temperature and higher CO_2_ concentration was a significantly shorter lag phase [7].

Finally, the winemaking process also influences the OTA contamination of the wine. Previously, a greater risk of OTA contamination was discussed for red wines. This fact can be explained by the different winemaking processes for white and red wines. White wines are pressed immediately after the harvest, whereas the grapes for red wines are first crushed and must be macerated for several days. During this time, OTA synthesis and its transition from the grapes to the must take place [12,74].

## 4. In Field Prevention of OTA Contamination in Wine

The problem of contamination of grapes with OTA begins in the vineyard, so the first preventive measures should be carried out there [10]. Certainly, good agricultural practice is necessary and could be helpful in prevention. The practices used to reduce fungal diseases in the vineyard, such as an optimal ratio of leaves to bunches, good ventilation of the bunches, or an optimal distance between bunches, could help prevent the appearance of OTA in wine [75]. As already mentioned, the critical period is between the beginning of the ripening period and the harvest. Some authors suggest that the critical period should be extended from early veraison to the beginning of vinification [5,10,44,45,74]. It is very important not to irrigate the vineyard too much to avoid bursting of the berries, which is especially important in the later stages of veraison. In addition, the harvest should be well planned, especially in regions where the risk of OTA contamination is higher. The time between harvest and vinification should be as short as possible [5,12,30,41,71,74,75].

The control of insects, especially *Lobesia botrana*, can contribute significantly to reducing the risk of contamination [71,76]. In a trial conducted in Sicily in 2006, the amount of OTA recovered from grapes infected with *L. botrana* was significantly higher than in uninfected grapes: 20 µg L^−1^ in infected grapes and 0.04 µg L^−1^ in uninfected grapes [65,76]. In another trial in which biological control agents of *Lobesia botrana* were used in the field for two years, there was an 80% reduction in the OTA content of the grapes in the year in which the meteorological conditions were favorable for insect infestation [68].

## 5. Biocontrol of OTA Occurrence in Wine

**Figure 2:**
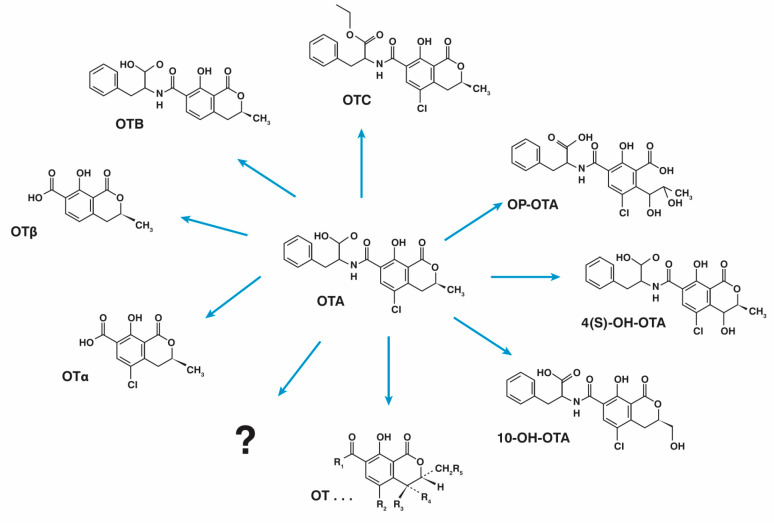
Possible metabolites of OTA as products of microbial detoxification methods (OTA—ochratoxin A; OTB—ochratoxin B; OTC—ochratoxin C; OTα—ochratoxin α; OTβ—ochratoxin β; OP-OTA—lactone-opened ochratoxin A; 4(S)-OH OTA—epimer of 4-hydroxyochratoxin A (4-OH OTA); 10-OH OTA—10-hydroxyochratoxin A; OT…—metabolites that remain to be characterized; ?—unknown metabolites).

As an alternative to chemical and physical OTA detoxification methods, there is now a growing interest in the use of biological methods, i.e., microorganisms involved in food fermentation, as biological agents for OTA control and detoxification. These biological methods are based on non-toxic bacteria, yeasts, and even fungi and other microorganisms as main components. Biological methods have many advantages, such as low cost and minimal side effects, and with the increasing awareness of environmental protection and food safety, they are the best representatives of the methods with lower environmental impact. They are widely used to reduce the concentration of OTA in food, grain, and feed [16,77]. Biological methods can be categorized into three groups based on the mechanisms of action: (1) inhibition of fungi responsible for OTA biosynthesis, (2) adsorption of OTA, and (3) degradation or detoxification of OTA in contaminated matrices [78]. Microbiological detoxification methods are defined as methods using microorganisms and/or enzymes that can metabolize, destroy, or deactivate toxins to stable, less toxic, or harmless compounds [79]. An example of microbial OTA detoxification metabolites is shown in Figure 2.

**Figure 3:**
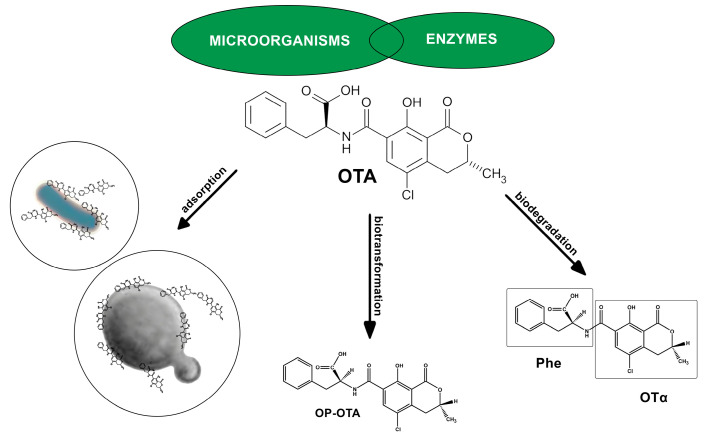
Schematic representation of mechanisms of microbiological control of OTA (adsorption—OTA bounded on cell wall surfaces of bacteria/yeast; biotransformation—hydrolysis of the lactone ring of OTA; biodegradation—hydrolysis of the peptide bond of OTA).

Biological agents and their associated enzymes allow a specific, probably irreversible, environmentally friendly, and effective approach with minimal impact on the sensory and nutritional quality of food compared to chemical methods used to reduce mycotoxin concentration [16,80,81]. Yeasts, especially *Saccharomyces* species and lactic acid bacteria (LAB), are mainly used as part of the natural microbial population in spontaneous food fermentation and as starter cultures in the food and beverage industry [74,82,83,84]. The microbial degradation of OTA could be mediated by the hydrolysis of the amide bond to the non-toxic compounds L-β-phenylalanine and OTα [84,85,86] or by hydrolysis of the lactone ring [85,86,87,88,89], while a proposed model for mycotoxin binding consists of two processes: binding (adsorption) and release (desorption) to/from the binding sites on the surface of the microorganism [89,90,91,92,93]. It has been shown that both viable and dead cells of microorganisms bind mycotoxins. Carbohydrate and/or protein components of the cell wall play an important role in binding mycotoxins; the binding efficiency depends on the strain of the microorganism, the amount of mycotoxin, the environmental conditions (pH), and the stability of the microorganism–mycotoxin complex [74,84,94,95,96]. Many microorganisms, including bacteria, yeasts, filamentous fungi, and protozoa, can remove, reduce, or biodegrade OTA [82,84,88,89,91,93,97]. Among these potential microorganisms, oenological LAB and yeasts represent a unique group of biocontrol agents for the biodegradation, removal, or binding of OTA due to their role in winemaking (Figure 3) without affecting the organoleptic properties of wine [86,97,98,99].

### 5.1. Inhibition of the Fungi Responsible for OTA Biosynthesis

The production of OTA can be inhibited in different ways, depending on the type of microorganism. For example, many bacteria can produce various lipopeptides that inhibit OTA production by altering the cell structure or even destroying the cell structure of filamentous fungi, which leads to metabolic imbalance and inhibits spore germination but can also downregulate genes related to OTA synthesis [100,101]. Biocontrol can be used directly against ochratoxigenic molds. In peanut cultivation, contamination in the field was reduced when a non-aflatoxin-producing strain of *Aspergillus flavus* was inoculated [102]. In vitro experiments with OTA-producing *A. carbonarius* and two mutants, Dpsk and DveA, which cannot produce OTA, gave encouraging results [103]. Non-toxigenic strains were able to displace ochratoxigenic strains in liquid media, and the OTA concentration was reduced in all tested concentrations due to the presence of non-toxigenic strains. Even in the most unfavorable concentrations of the inoculum (10-fold higher concentration of the toxigenic strain), the OTA concentration was reduced by almost 30%. Similar results were obtained in grape berries, suggesting that the use of competitive, non-toxigenic *A. carbonarius* strains in vineyards could be an effective means of OTA control. In laboratory experiments, some yeast strains of *Saccharomyces cerevisiae* and *Kloeckera apiculate* showed inhibition of *A. carbonarius* growth and OTA synthesis on both liquid media and grape berries [104]. Yeast strains of other species, Candida intermedia, and *Lachancea thermotolerance* inhibited *A. carbonarius* growth and OTA production, both in vitro and on berries [105]. Although there is evidence that some yeasts or fungi could control the growth of *A. carbonarius* on grape berries, there is no large-scale product on the market based on biocontrol agents. Yeasts are being studied in more detail as biocontrol agents for the removal of OTA in winemaking and are discussed below.

### 5.2. Effects of OTA on Bacteria and Yeasts

#### 5.2.1. Effect of OTA on Bacteria

The effect of OTA on LAB is also important, but the few published studies suggest that bacteria, including LAB, are microorganisms that are resistant to OTA. Little is still known about the effects of mycotoxins on LAB, as the toxicity thresholds reported by different authors vary. Therefore, Piotrowska and Zakowska [106] conducted a study in which the sensitivity of LAB to the effects of different OTA concentrations was investigated. In the range of 0.1 to 10 µg, only one LAB was sensitive to OTA, while other LAB strains, with few exceptions (*Lactobacillus acidophilus* Ind1 and *L. acidophilus* H-1), showed no sensitivity to OTA at concentrations of 0.1 to 5 µg. Their growth was inhibited by OTA at 5 µg. At the same time, high concentrations of OTA (10 µg) proved to be growth inhibitory for most strains. Only a few strains remained unaffected by the OTA concentrations (*L. acidophilus* B, *L. acidophilus* CH-5, and *L. rhamnosus* GG). The results obtained show that OTA has a negative effect on the growth of LAB, but these results differ from the results of [107], who showed that OTA at a concentration of 20 ppm does not inhibit the growth of *L. plantarum* and *L. casei*. According to [108], two concentrations of OTA (2 and 4 µg/mL) had minimal effect on the growth of *L. plantarum* B, with visible activity only during the lag phase, which was extended by two hours.

#### 5.2.2. Effects of OTA on Yeasts

Much is known about the use of yeasts for biocontrol, degradation, removal, or binding of OTA [56,84,98,109]. However, little is known about the effects of mycotoxins as stress factors on yeasts, their morphology, their growth parameters, their metabolic activity, and their effects on fermentation [98]. Therefore, it is possible that OTA can inhibit the enzymes of ethanol fermentation, impair cell growth, and induce oxidative stress. The presence of OTA had an impact on the viability of the *Saccharomyces* strains studied. In particular, lower concentrations of OTA (2 µg/mL) had an inhibitory effect on the cell growth of both *S. cerevisiae* and *S. uvarum*. Although the viability of *S. cerevisiae* was not significantly affected, the cell diameter changed considerably. Since OTA had a major effect on the cell diameter and viability of *S. uvarum*, it is clear that the effect of OTA is strain-specific [110]. In the study by Jakopović et al. [98], ethanol synthesis by the yeast *S. uvarum* was slowed by 4 µg/mL OTA. Freire et al. [111] investigated the effects of 10, 20, and 30 µg/L OTA on three different yeast strains of *S. cerevisiae* and obtained similar results. The presence of ochratoxin A during fermentation did not affect the growth of the *S. cerevisiae* strains, but some differences were observed. For example, one of the *S. cerevisiae* strains tested showed a lower growth rate and a longer lag phase in the presence of OTA than the other two strains tested. In addition, the same *S. cerevisiae* strain had a longer exponential and stationary phase. Despite all this, the maximum population of the yeasts studied was not affected by the presence of different concentrations of OTA in the fermentation medium.

### 5.3. Adsorption of OTA

Ample evidence of the beneficial effect of LABs and their additional probiotic properties gives them significant potential for their application in food and feed, and they are also good candidates as mycotoxin-biotransforming bacteria [84,89,112,113,114]. The ability to reduce the amount of OTA is common in LAB but varies by species and bacterial strain.

After studying the effects of OTA on LAB, Piotrowska and Zakowska [106] investigated the phenomenon of OTA elimination by LAB. The study of the average values of LAB species showed that *Lactobacillus acidophilus*, *L. rhamnosus*, *L. sanfranciscens,* and *L. plantarum* have the greatest ability to remove OTA. However, analysis of the values obtained for specific strains within species revealed significant differences, again confirming that the ability to bind OTA is strain-specific. Del Prete et al. [115] analyzed 15 strains belonging to five relevant oenological LAB species for their sensitivity to ochratoxin A and their ability to remove this toxin from liquid media. The amount of OTA removed during bacterial growth was found to be between 8 and 28%, with *Oenococcus oeni* being the most effective strain, reducing OTA by 28%. OTA was not degraded by cell-free extracts of oenological LAB strains, and no degradation products were detected, suggesting that the removal of OTA by oenological LAB is a binding process. Mateo et al. have shown that several strains of *O. oeni* reduce OTA levels by 50–70%, while *Lactobacillus acidophilus* reduces OTA in the broth medium by ≥98%, while heat-treated LAB cells remove small amounts of OTA (less than 11%) [91]. In contrast, a study by Piotrowska [116] showed that heat-inactivated *Lactobacillus* cells reduced OTA (46.2–59.8%) more efficiently than live cells (16.9–35%). Shukla et al. [117] observed that the amount of OTA in red wine samples was reduced by 90% when treated with heat-treated *B. subtilis* cells. The results obtained by several researchers have shown that cell wall components such as peptidoglycan and polysaccharides play an important role in the binding of OTA. Due to changes in the cell surface, mycotoxin binding is permanent when the LAB cells are dead (heat or acid-treated), while the living bacteria can release part of the mycotoxin content over time [84,92,106,114,118,119]. The higher OTA adsorption by dead cells compared to viable cells can be explained by changes in the bacterial cell wall or by the hydrophobic nature of the bacterial cell wall. Thermal or acidic treatment of the cells leads to denaturation of the proteins and increased permeability of the outer layers of the cell wall. As a result, a larger number of active sites are formed, which are responsible for the absorption of various compounds [116]. Although the results of several researchers have shown that LAB cells can adsorb OTA and it is removed or reduced from the medium in the absence of metabolically active cells, the results of other researchers have shown that metabolic degradation by hydrolysis of the amide bond or by hydrolysis of the lactone ring may be the mechanism responsible for OTA removal [77,84,88,89,91,114].

Table 2 summarizes the microorganisms able to adsorb more than 75% of the OTA present in the media.

### 5.4. Degradation of OTA

In their study, Rodriguez et al. [120] investigated the ability to degrade OTA using *Pseudomonas putida* and various bacteria of the genera *Rhodococcus* and *Brevibacterium.* After the growth of the bacteria in a liquid synthetic medium and the addition of OTA (~10 µg/L), a small decrease in OTA concentration (8–28%) was observed in the supernatants of *Rhodococcus* and *P. putida* strains with no evidence of degradation products [120]. This suggests that OTA is not degraded but adsorbed by the cells, as previously described [114,115,121,122,123]. On the other hand, analysis of the supernatant of *B. casei* RM101 showed the complete absence of OTA [123]. Since *Brevibacterium* species differ from other bacteria in their ability to metabolize compounds with heterocyclic and polycyclic ring structures (a property also exhibited by fungi), further analyses were performed [120]. *Brevibacterium* strains can completely degrade OTA, even at concentrations of up to 40 mg/L, a concentration 1000 times greater than that commonly found in foodstuffs. Therefore, it is clear that OTA degradation is characteristic of the genus *Brevibacterium*. Abrunhosa and coworkers [89] tested the ability of 19 different LAB strains for wine to degrade OTA. *Pediococcus parvulus* UTAD 473 showed the most promising results: it converted 90% of OTA into less toxic OTα. Rodrigues et al. [124] reported that OTα is non-toxic or at least 500 times less toxic than OTA.

Table 3 lists the microorganisms for which OTA removal by degradation of more than 75% was reported.

#### Enzymatic Degradation of OTA

Enzymes are mainly derived from the production of specific microbial strains and are an important component of the biodegradation mechanisms of OTA. The enzymes produced can be both extracellular and intracellular [125,126,127], and the most common types of OTA biodegradation enzymes are mainly carboxypeptidase and amidase [16]. The review by [81] focuses on the biotransformation of mycotoxins with purified enzymes isolated from bacteria, fungi, and plants, whose activity has been validated by in vitro and in vivo tests. According to scientific studies, crude and purified enzymes are involved in the transformation of OTA. These enzymes include several classes of carboxypeptidases: carboxypeptidase A (CPA), carboxypeptidase B (CPB), and carboxypeptidase Y (CPY); metalloenzymes derived from atoxigenic *Aspergillus niger* strains, as well as lipase A, protease A, ochratoxinase, and amidases that hydrolyze the amide bond of OTA to generate OTα and phenylalanine [86,128,129,130,131,132,133,134,135,136,137]. The first report on the biodetoxification of OTA dates back to 1969 [138] and used bovine pancreatic carboxypeptidase A (CPA) to cleave OTA into OTα. Dobritzsch et al. [132] reported that purified recombinant ochratoxinase was about 600 times more effective than CPA, with optimal OTA degradation activity at 66 °C and pH 6. In the studies using CPY isolated from *S. cerevisiae*, it was shown that only 52% of OTA was converted to OTα at 37 °C and pH 5.6 [86,129] have shown that the use of commercial hydrolases, such as lipase preparations from *A. niger*, is also capable of hydrolyzing OTA to OTα and phenylalanine. Abrunhosa et al. [130] reported that Ancex, a crude enzyme isolated from *A. niger*, was able to degrade 99.8% of OTA (1 µg/mL) to OTα after 25 h of incubation at pH 7.5 and 37 °C. Commercially purified enzymes such as protease A and pancreatin were not able to degrade as much OTA (87.3 and 43.4%, respectively). Another example is Prolyve PAC, which was only able to degrade 3% of OTA (1 µg/mL) to OTα after 25 h of incubation at pH 3 and 37 °C [124]. While Bejaoui et al. (2006) [139] reported that OTα was further degraded to unknown products, several authors reported that OTA could be directly degraded to unknown products [99,140,141]. Since not every enzymatic reaction leads to real detoxification, because the metabolized mycotoxin can take on greater toxic properties than the parent compound, it is necessary to identify and characterize the degrading enzymes and understand the mechanism of degradation and test the toxicity of OTA by-products [16,137,142].

In Table 4 are reported enzymes produced by microorganisms able to degrade the OTA.

The OTA-degrading enzymes produced by microorganisms are listed in Table 4.

## 6. Conclusions

Climate change poses various risks for plant cultivation; for example, it can promote the occurrence and concentration of OTA in wine. In order to avoid or limit this, prevention strategies that are sustainable for the environment must be developed. Increasing fungal resistance and the challenges associated with conventional systems require the development of strategies that allow both efficient biocontrol of ochratoxigenic strains and rapid elimination of OTA from the wine with short processing times and negligible impact on quality.

This review shows that biological control is an important, environmentally friendly, and efficient tool for controlling the presence of OTA in wine. Although some yeasts and atoxigenic strains of *A. carbonarius* have been shown to be effective in controlling the growth of ochratoxigenic fungi and are able to inhibit OTA synthesis, no biological control agent is yet widely and commercially used in vineyards for OTA prevention. Regardless of the effectiveness of preventive measures, human exposure to this mycotoxin can also be minimized by decontamination and detoxification strategies once OTA is detected in wine. Various biological decontamination and detoxification methods have been proposed to limit the contamination of wine with OTA. In particular, the use of different microorganisms, such as LAB and *S. cerevisiae*, and microbial enzymes has shown that over 90% of OTA can be efficiently removed from wine. The effects of OTA degradation on the organoleptic properties and taste of wine should be investigated before the large-scale application of this method.

Further research is needed on microorganisms that can prevent the occurrence of OTA or safely remove it from the wine without affecting the organoleptic properties of the wine. In particular, research into biocontrol agents that can control the presence and growth of ochratoxigenic strains in vineyards is desirable.

## Figures and Tables

**Table 2:** An overview of the microorganisms reported to remove over 75% of OTA from media by adsorption.

Microorganism	Strain	OTA Removal (%)	Source
*Saccharomyces cerevisiae*	-	82.8–85.1 in grape	116
	-	73–90	84,110,111
	BS	75–77	116
	EC 118	29–95	84
	Y28	81.87	83
	TP5 TT173	79–100/ 47–86	109
*Candida intermedia*	-	>80	78
*Debaryomyces hansenii*	-	>98	84
*Bacillus subtilis*	-	78	117
*Gluconobacter oxydans*	-	>80	113

**Table 3:** An overview of the microorganisms reported to degrade more than 75% of the OTA present in the media.

Microorganism	Strain	OTA Degradation Rate (%)	Source
*Saccharomyces cerevisiae*		83–85	78, 84
*Candida intermedia*		>80	84
*Debaryomyces hansenii*		>98	84
*Bacillus subtilis*		78	84, 117
*Brevibacterium casei*	RM101	100	120
	DSM 20657 T DSM 9657 ND DSM 20658 RM101	100	120
*Brevibacterium linens*	DSM 20425 T	100	120
*Brevibacterium iodinum*	DSM20626T	100	120
*Brevibacterium epidermidis*	DSM 20660T	100	120
*Lactobacillus kefiri*	KFLM3	81	121
*Pediococcus parvulus*	UTAD 168	89	89
	UTAD 333	97	89
	UTAD 334	94	89
	UTAD 335	98	89
	UTAD 473	100 ± 0	89
*Aspergillus niger*		>90%	124
*Rhizopus* sp.		100	124
*Trichosporon* *mycotoxinivorans*	MTV	100	124

**Table 4:** Microbial enzymes able to degrade OTA, species that produce them, and degradation products.

Microorganism	Enzyme	Degradation Product(s)	Source
*Aspergillus niger*	Lipase	Acid L-β-phenylalanine and (PHE) and OTα	129
	Protease A (acid protease)	OTα	86
	Prolyve PAC (acid protease)	OTα	86
*Saccharomyces cerevisiae*	Carboxypeptidase Y (CPY)	OTα	86
*Stenotrophomonas acidaminiphila* CW117	Amido hydrolase ADH3	OTα	137

## Data Availability

Data sharing not applicable, no new data is generated.
